# Supplementary material for: Assessment of heavy metal pollution in surface sediments of the Chishui River Basin, China
Source: PLoS One. 2022 Feb 9;17(2):e0260901. doi: 10.1371/journal.pone.0260901 (PMC8827479; doi:10.1371/journal.pone.0260901)
Supplement: S1 File — (DOCX) [file pone.0260901.s001.docx]

**Highlights**

- Sediment concentrations of heavy metals were investigated in the Chishui River Basin.
- Metal contamination was in the order of Zn > Cu > As > Cd > Hg.
- Contamination indexes identified Hg as the primary pollutant.
- Pearson’s correlation determined that Cu, Zn, and As had a similar source.
