# Supplementary material for: Assessment of heavy metal pollution in surface sediments of the Chishui River Basin, China
Source: PLoS One. 2022 Feb 9;17(2):e0260901. doi: 10.1371/journal.pone.0260901 (PMC8827479; doi:10.1371/journal.pone.0260901)
Supplement: Attachment — Submitted filename: PONE-D-21-22502_reviewer.pdf [file pone.0260901.s002.pdf]

PLOS ONE

Assessment of Heavy Metal Pollution in Surface Sediments of the Chishui River Basin, China

--Manuscript Draft--

**Manuscript Number:**	PONE-D-21-22502
**Article Type:**	Research Article
**Full Title:**	Assessment of Heavy Metal Pollution in Surface Sediments of the Chishui River Basin, China
**Short Title:**	Assessment of Heavy Metal Pollution in Surface Sediments
**Corresponding Author:**	fanxi li<br>Guizhou University<br>Guiyang, CHINA
**Keywords:**	Chishui River; Sediments; Heavy metals; Contamination; Ecological risk
**Abstract:**	Accumulated heavy metals in surface sediments are released into the aquatic environment, causing secondary contamination of the hydrosphere, and increasing the risks to human health. To evaluate the pollution characteristics of heavy metals in the sediments of the Chishui River Basin, in the present study, the concentrations of five heavy metals in surface sediments of the Chishui River Basin in China were investigated using the geo-accumulation, pollution load, and potential ecological risk indexes. These indexes evaluated the degree of contamination and the influence of human activities on heavy metal levels in the basin. Cu, Zn, Cd, Hg, and As were found at concentrations of 5.12–120.40, 36.01–219.31, 0.03–1.28, 0.01–1.18, and 1.56–11.59 mg kg –1 , respectively, with mean values of 37.43, 91.92, 0.25, 0.07, and 5.16 mg kg –1 , respectively, in the order Zn > Cu > As > Cd > Hg. The contamination indices revealed Hg as the principal pollutant based on the spatial distribution, while Pearson's correlation coefficients suggested that Cu, Zn, and As originated from a similar source. Hg had a different source from the other metals, whereas Cd originated from a different source compared with that of Zn, As, and Hg. This paper showed a Hg and Cd contamination in the Chishui River Basin.
**Order of Authors:**	fanxi li
**Additional Information:**	
**Question**	**Response**
**Financial Disclosure**<br><br>Enter a financial disclosure statement that describes the sources of funding for the work included in this submission. Review the submission guidelines for detailed requirements. View published research articles from *PLOS ONE* for specific examples.<br><br>This statement is required for submission and **will appear in the published article** if the submission is accepted. Please make sure it is accurate.	This study was funded by the Open Fund of the State Key Laboratory of Loess and Quaternary Geology [grant number SKLLQG2036], the Chinese Academy of Sciences Strategic Leading Science and Technology Project (Category B) [grant number XDB40000000], the Guizhou Institute of Technology High-level Talent Research Startup Funding Project [grant number 2019, 45], the Science and Technology Program of Guizhou Province (Guizhou Science and Technology Cooperation Support [grant number 2018, 20101]), and the Science and Technology Foundation of Water Resources Department of Guizhou Province [grant number KT201508].

Powered by Editorial Manager® and ProduXion Manager® from Aries Systems Corporation

**Unfunded studies**<br>Enter: *The author(s) received no specific funding for this work.*<br><br>**Funded studies**<br>Enter a statement with the following details:<br>• Initials of the authors who received each award<br>• Grant numbers awarded to each author<br>• The full name of each funder<br>• URL of each funder website<br>• Did the sponsors or funders play any role in the study design, data collection and analysis, decision to publish, or preparation of the manuscript?<br>• **NO** - Include this sentence at the end of your statement: *The funders had no role in study design, data collection and analysis, decision to publish, or preparation of the manuscript.*<br>• **YES** - Specify the role(s) played.<br><br>* typeset	
**Competing Interests**<br><br>Use the instructions below to enter a competing interest statement for this submission. On behalf of all authors, disclose any competing interests that could be perceived to bias this work—acknowledging all financial support and any other relevant financial or non-financial competing interests.<br><br>This statement is required for submission and **will appear in the published article** if the submission is accepted. Please make sure it is accurate and that any funding sources listed in your Funding Information later in the submission form are also declared in your Financial Disclosure statement.<br><br>View published research articles from *PLOS ONE* for specific examples.	The authors have declared that no competing interests exist.

**NO authors have competing interests**<br><br>Enter: *The authors have declared that no competing interests exist.*<br><br>**Authors with competing interests**<br><br>Enter competing interest details beginning with this statement:<br><br>*I have read the journal's policy and the authors of this manuscript have the following competing interests: [insert competing interests here]*<br><br>* typeset	
**Ethics Statement**<br><br>Enter an ethics statement for this submission. This statement is required if the study involved:<br><br>• Human participants<br>• Human specimens or tissue<br>• Vertebrate animals or cephalopods<br>• Vertebrate embryos or tissues<br>• Field research<br><br>Write "N/A" if the submission does not require an ethics statement.<br><br>General guidance is provided below. Consult the submission guidelines for detailed instructions. **Make sure that all information entered here is included in the Methods section of the manuscript.**	N/A

**Format for specific study types**<br><br>**Human Subject Research (involving human participants and/or tissue)**<br>• Give the name of the institutional review board or ethics committee that approved the study<br>• Include the approval number and/or a statement indicating approval of this research<br>• Indicate the form of consent obtained (written/oral) or the reason that consent was not obtained (e.g. the data were analyzed anonymously)<br><br>**Animal Research (involving vertebrate animals, embryos or tissues)**<br>• Provide the name of the Institutional Animal Care and Use Committee (IACUC) or other relevant ethics board that reviewed the study protocol, and indicate whether they approved this research or granted a formal waiver of ethical approval<br>• Include an approval number if one was obtained<br>• If the study involved *non-human primates*, add *additional details* about animal welfare and steps taken to ameliorate suffering<br>• If anesthesia, euthanasia, or any kind of animal sacrifice is part of the study, include briefly which substances and/or methods were applied<br><br>**Field Research**<br><br>Include the following details if this study involves the collection of plant, animal, or other materials from a natural setting:<br>• Field permit number<br>• Name of the institution or relevant body that granted permission	
**Data Availability**<br><br>Authors are required to make all data underlying the findings described fully available, without restriction, and from the time of publication. PLOS allows rare exceptions to address legal and ethical concerns. See the PLOS Data Policy and FAQ for detailed information.	Yes - all data are fully available without restriction

A Data Availability Statement describing where the data can be found is required at submission. Your answers to this question constitute the Data Availability Statement and **will be published in the article**, if accepted.<br><br>**Important:** Stating 'data available on request from the author' is not sufficient. If your data are only available upon request, select 'No' for the first question and explain your exceptional situation in the text box.<br><br>Do the authors confirm that all data underlying the findings described in their manuscript are fully available without restriction?	
**Describe where the data may be found in full sentences. If you are copying our sample text, replace any instances of XXX with the appropriate details.**<br><br>• If the data are **held or will be held in a public repository**, include URLs, accession numbers or DOIs. If this information will only be available after acceptance, indicate this by ticking the box below. For example: *All XXX files are available from the XXX database (accession number(s) XXX, XXX.)*.<br>• If the data are all contained **within the manuscript and/or Supporting Information files**, enter the following: *All relevant data are within the manuscript and its Supporting Information files.*<br>• If neither of these applies but you are able to provide **details of access elsewhere**, with or without limitations, please do so. For example:<br><br>*Data cannot be shared publicly because of [XXX]. Data are available from the XXX Institutional Data Access / Ethics Committee (contact via XXX) for researchers who meet the criteria for access to confidential data.*<br><br>*The data underlying the results presented in the study are available from (include the name of the third party*	All relevant data are within the manuscript and its Supporting Information files.

and contact information or URL).<br>• This text is appropriate if the data are owned by a third party and authors do not have permission to share the data.<br><br>* typeset	
Additional data availability information:	

Manuscript Click here to access/download;Manuscript;Manuscript.docx

Assessment of Heavy Metal Pollution in Surface Sediments of the Chishui River Basin, China

Fanxi Li[a], Xia Yu[b*], Jiemei Lv[b], Qixin Wu[a,c], Yanling An[b*]

[a] Key Laboratory of Karst Environment and Geohazard Prevention, Guizhou University, Guiyang, Guizhou Province 550025, China

[b] The College of Resources and Environmental Engineering, Guizhou Institute of Technology, Guiyang, Guizhou province 550003, China

[c] The College of Resources and Environmental Engineering, Guizhou University, Guiyang, Guizhou Province 550025, China

ABSTRACT

Accumulated heavy metals in surface sediments are released into the aquatic environment, causing secondary contamination of the hydrosphere, and increasing the risks to human health. To evaluate the pollution characteristics of heavy metals in the sediments of the Chishui River Basin, in the present study, the concentrations of five heavy metals in surface sediments of the Chishui River Basin in China were investigated using the geo-accumulation, pollution load, and potential ecological risk indexes. These indexes evaluated the degree of contamination and the influence of human activities on heavy metal levels in the basin. Cu, Zn, Cd, Hg, and As were found at concentrations of 5.12–120.40, 36.01–219.31, 0.03–1.28, 0.01–1.18, and 1.56–11.59 mg kg^{-1}, respectively, with mean values of 37.43, 91.92, 0.25, 0.07, and 5.16 mg kg^{-1}, respectively, in the order Zn > Cu > As > Cd > Hg. The contamination indices revealed

*Corresponding author: Guizhou Institute of Technology, Guiyang, 550003, China; E-mail: anyanling@git.edu.cn(yanling an); yuxia@git.edu.cn(xia yu)

Hg as the principal pollutant based on the spatial distribution, while Pearson's correlation coefficients suggested that Cu, Zn, and As originated from a similar source. Hg had a different source from the other metals, whereas Cd originated from a different source compared with that of Zn, As, and Hg. This paper showed a Hg and Cd contamination in the Chishui River Basin.

Keywords: Chishui River; Sediments; Heavy metals; Contamination; Ecological risk

1. Introduction

Contaminants containing high concentrations of heavy metals continue to be discharged into aquatic systems. These metals are often deposited on the bottom of such systems via precipitation and flocculation, thereby transforming the associated sediments into heavy metal repositories (Bartoli et al., 2012; Fang et al., 2013; Malvandi, 2017). Due to their non-degradability, toxicity, and resistance to metabolization (Fabio et al., 2021; Suresh et al., 2012), heavy metals in sediments can harm aquatic organisms, as well as human health, through bioaccumulation and bioamplification (Saiful et al., 2015). In aquatic ecosystems, the proportion of heavy metals present as dissolved ions is low because most metals are deposited in the associated sediments (Awadh and Kong, 2021; Fabio et al., 2021; Herath et al., 2018). However, heavy metals in sediments can be released and discharged into aquatic systems via changes in water conditions, such as the hydrodynamics, temperature, and pH, causing secondary pollution (Zoumis et al., 2001).

Therefore, the principal objective of this study was to determine the concentrations

of f[illegible] metals, i.e., Cu, Zn, Cd, Hg, and As, in the surface sediments in the Chishui River, China. The extent of pollution due to these metals was characterized using the geo-accumulation, pollution load (PLI), and potential ecological risk (RI) indexes. The findings of this study may be useful for future investigations on heavy metals in river ecosystems, heavy metal pollution management, and policy formulation.

The Chishui River (104°45′–106°51′ E; 27°20′–28°50′ N) in southwest China is located at the transitional zone between the Yunnan-Guizhou Plateau and the Sichuan Basin (Fig. 1). Although no dam has been constructed on the river, it is an important tributary along the upper reaches of the Yangtze River. It flows through 13 counties in three provinces, with a mainstream length of 436.5 km, a natural head of 1,580 m, and a basin area of 1.91×10^4 km^2 (Huang, 2003). Assessment of sediment heavy metal pollution is critical for the ecological protection of the Chishui River. The average water volume in the Chishui River in the past few years has been reported to be 9.74×10^9 m^3 while the average flow rate into estuaries is approximately 309 m^3/s (Qiu et al., [illegible]3). The Chishui River and other tributaries are naturally connected to the Yangtze River, and the associated hydrological processes provide suitable breeding conditions for migratory fish species (Wu et al., 2010). According to Wu (2010), among the 135 fish species reported in the Chishui River, approximately 40 were considered endemic to the upper Yangtze in 2007. Therefore, the survival of these species is threatened by the input of contaminants, such as heavy metals and organic pollutants, into the ecosystem.

Previous stu[illegible]focused on the development and protection of cultural (Wang,

2011), touristic (Ren and Huang, [illegible]d natural (Cao, 2000; Huang, 2003; Wu et al., 2010; Zhen-li, 2003) resources along the Chishui River. Among the few available studies on the physicochemical properties of the Chishui River, Wu (2001) systematically identified the major components of the river water, as well as the background composition and characteristics of 15 trace elements in the raw and filtered water, suspended solids, sediments, and aquatic organisms. Additionally, Zou et al. (2010) used the total phosphorus (TP), suspended solids (SS), and pH values to evaluate the water quality in the central section of the Chishui River while Ji et al. (2012) analyzed variations in the water quality in Maotai using the permanganate index, biochemical oxygen demand (BOD_5), and ammonia as indicators. Furthermore, Jiang et al. (2013) studied the spatial distribution of the water quality in the Chishui River during the dry season. Lv et al. (2013) and Luo et al. (2014) also investigated the compositional variations at the midstream and downstream points, respectively, along the Chishui River during the dry season. Thus, limited studies have examined heavy metal pollution and their spatial distributions in the sediments along the entirety of the Chishui River Basin.

2. Materials and methods

2.1. Sediment sample collection

Samples were collected during a dry period in December 2012. At each site, sediment layers from the top 0–10 cm were collected from v[illegible]nts, which were mixed to produce a composite sample. Sediment samples were collected from 32 stations (Fig. 1) throughout the Chishui River Basin. The samples were sealed in plastic

bags, stored at 4 ℃, and transported to the laboratory for heavy metal analyses. In the laboratory, the sediments were spread on plastic films; stones, branches, and other plant materials were removed. The samples were then stored under dry conditions at room temperature. After gently rolling using a wooden stick according to the four-diagonal method (Luo et al., 2014), samples smaller than 200-mesh were collected and stored in polyethylene bags for testing.

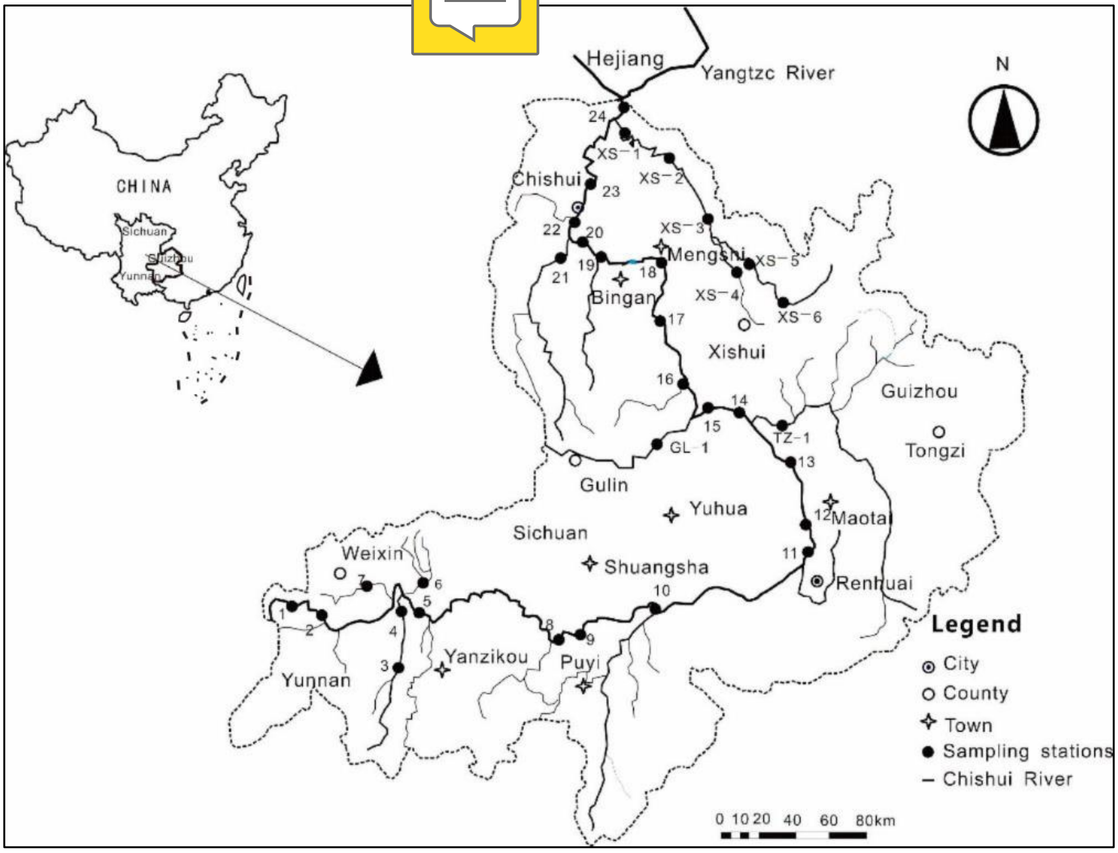

Fig. 1. Map of the sampling stations in the Chishui River Basin, China.

2.2. Heavy metal analyses

All analyses were performed at the Key Laboratory of Karst Environment and Geohazard Prevention, Ministry of Education, Guizhou University. An HNO_3-HF mixture was added to approximately 0.05 g of sediment in a Teflon vessel, and the mixture was subjected to digestion at 140 °C on a hot plate. The sample was then

removed from the acid mixture after it appeared white or light-colored. Cu, Zn, and Cd concentrations in the sediments were determined using flame atomic absorption spectrometry (AAS; Contr AA 700, Germany) while Hg and As concentrations were measured via cold vapor AAS using the digested sample (0.3 g; GB/T 22105.1-2008). To ensure accuracy and precision of the measurements, stream sediment (GBW07309) and soil (GBW07401) standards were used for quality control. Samples were analyzed in triplicate, and the relative standard deviations were < 5 %. Ultrapure water was used for sample preparation for all tests, and all reagents were of guaranteed quality.

The SPSS Statistics software (version 25.0, IBM) was used for analyzing the correlation matrix of the heavy metals present in the surface sediments. Microsoft Excel (version 2019) was used for statistical analysis of the test data; a p-value of < 0.05 was taken as significant.

2.3. Sediment pollution a[illegible] methods

2.3.1. Geo-accumulation index

I_{geo} is an effective parameter for assessing heavy metal pollution levels in sediments. It can be obtained using the following equation:

$$I_{geo} = \log_2[\,C_i/(1.5 \times B_i)], \qquad (1)$$

where C_i represents the concentration of the heavy metal n and B_i represents the background level (mg kg^{-1}). A factor of 1.5 was used for lithological variations in the background value, based on previously reported values for shales (Muller, 1969). The I_{geo} classes established by Muller (1969) for heavy metal pollution are presented in Table 1.

Table 1 Congruent relationships between metal I_{geo} values and pollution levels (from Miller, 1969).

I_{geo}	Contamination level
$I_{geo} \leq 0$	Unpolluted
$0 < I_{geo} \leq 1$	Unpolluted to moderately polluted
$1 < I_{geo} \leq 2$	Moderately polluted
$2 < I_{geo} \leq 3$	Moderately to heavily polluted
$3 < I_{geo} \leq 4$	Heavily polluted
$4 < I_{geo} \leq 5$	Heavily to extremely polluted
$I_{geo} > 5$	Extremely polluted

2.3.2. Pollution load index

The pollution load index (PLI), proposed by Tomlinson (1980), is used to evaluate the overall toxicity status of a sample associated with heavy metals (Kükrer et al., 2014). It reflects the changing trends in heavy metal pollution in time and space. The PLI can be calculated using the followed equations:

$$\mathrm{CF_i} = C_i/B_i, \quad (2)$$

$$\mathrm{PLI_{site}} = \sqrt[\mathrm{n}]{\mathrm{CF_1} \times \mathrm{CF_2} \times \cdots\cdots \times \mathrm{CF_n}}, \text{ and} \quad (3)$$

$$\mathrm{PLI_{zone}} = \sqrt[\mathrm{m}]{\mathrm{PLI_1} \times \mathrm{PLI_2} \times \cdots\cdots \times \mathrm{PLI_m}}, \quad (4)$$

where $\mathrm{CF_i}$ represents a pollution coefficient, $\mathrm{C_i}$ represents the measured concentration of a metal in the sediments, $\mathrm{B_i}$ represents the background value of a heavy metal, n represents the number of heavy metals investigated, and m represents the number of sampling sites. Table 2 lists the classes of the PLI and their corresponding contamination levels.

Table 2 Pollution grading standards based on the pollution load index (PLI).

PLI	Contamination level
PLI < 1.0	No contamination
1.0 ≤ PLI < 2.0	Moderately contaminated
2.0 ≤ PLI < 3.0	Considerably contaminated
PLI ≥ 3.0	Strongly contaminated

2.3.3. Potential ecological risk index

The potential ecological risk index (RI) is used to assess the level of heavy metal pollution in sedimentary environments (Hakanson, 1980). This is a widely utilized advanced index, which investigates the heavy metal content, the ecological effect of heavy metals, environmental benefits, and toxicology. The potential ecological risk factor, E_r^i, can be calculated as follows:

$$E_r^i = T_r^i \times \frac{C^i}{C_n^i}, \quad (5)$$

where T_r^i represents the toxic-response factor of a given substance, C^i represents the concentration of metal *i* in the sediments, and C_n^i denotes the background value of metal *i*. According to the findings of Hakanson (1980), the toxic response factors for Zn, Cu, As, Cd, and Hg, are 1, 5, 10, 30, and 40, respectively.

The RI is calculated by summing the E_r^i values, as follows:

$$RI = \sum_1^n E_r^i. \quad (6)$$

Background values (BV) for Hg, Cd, As, Cu, and Zn are 0.034, 0.15,7.6, 21.5, and 73.6 mg kg^{-1}, respectively (Alan, 1993; Wedepohl and Merian, 1991). Table 3 lists the derived five categories of E_r^i and four classes of RI.

Table 3 Indexes of the potential ecological risk and grades.

Potential Ecological risk factor (E_r^i)	Ecological risk level	Potential Ecological risk index (RI)	Ecological risk level
Er ＜40	Low potential ecological risk	RI ≤ 150	Low potential ecological risk
40 ≤ Er ＜80	Moderate potential ecological risk	150 ≤ RI ＜300	Moderate potential ecological risk
80 ≤ Er ＜160	Considerable potential ecological risk	300 ≤ RI ＜600	Considerable potential ecologic risk
160 ≤ Er ＜320	High potential ecological risk	600 ≤ RI	Very high ecological risk
320 ≤ Er	Very high ecological risk		

3. Results and discussion

3.1. Descriptive statistics for heavy metals

Concentration levels in sediments showed a variation with the distance from the start of the river(Fig.2). The heavy metal concentration level of upstream is higher than downstream, it indicated that there was no large-scale heavy metal pollution in downstream. Further, a point source pollution may appealed in tributaries as Tongzi River (TZ-1) was affected by As Pollution and Gulin River (GL-1) was polluted by Cd.

Table 4 presents statistical data based on the metal concentrations in the sediments

from the Chishui River Basin and background values. The sum of the heavy metal concentrations (Cu, Zn, Cd, Hg, and As) in the 32 samples varied between 51.92 and 314.10 mg kg^{-1}, yielding an average total concentration of 134.84 mg kg^{-1}.

The concentrations of Cu, Zn, Cd, Hg, and As in the samples ranged from 5.12–120.40, 36.06–219.33, 0.03–1.28, 0.01–1.18, and 1.57–11.59 mg kg^{-1}, respectively, with corresponding mean values of 37.43, 91.93, 0.25, 0.07, and 5.16 mg kg^{-1}. The sum of the Zn and Cu concentrations represented 95.94 % of the total heavy metal concentration, with the following order for the average sediment concentrations: Zn > Cu > As > Cd > Hg. The coefficient of variation values presented in Table 5 varied from 43 % for As to 280.37 % for Hg, producing the following sequence: Hg > Cd > Cu > Zn > As. These findings highlight the higher spatial variations for Hg, Cd, and Cu relative to Zn and As.

In this study, the Cu concentration in 65.63 % of the samples exceeded the BV. Overall, samples obtained from the upstream region showed higher Cu concentrations than those obtained from the middle and lower reaches of the Chishui River. The maximum Cu concentration (sample 7) was 5.6-fold higher than the BV while the minimum concentration (sample 19) was 24 % of the BV. This is because site 7 is in Weixin County, where industrial effluents and other pollutants from human activities are common. Similarly, the maximum Zn concentration measured in sample 12 was approximately three-fold higher than the BV while the minimum value obtained from XS-1 was 49 % of the BV. The concentrations of As in all samples were lower than those of Cu and Zn, but were higher than those of Cd and Hg. The highest As

concentration, obtained from TZ-1, was almost 1.5-fold higher than the BV while Cd associated with GL-1 was 8.53-fold greater than the BV. The high Cd levels in the GL-1 samples indicate severe Cd contamination in the Gulin River: the mining industry in this area discharges industrial wastewater into this river, and soils in the area are likely contaminated with Cd. The highest Hg concentration was found in sample 24 collected from Hejiang County, which is located at a site where the Chishui River flows into the Yangtze River; its value was 39.33-fold greater than that of the BV. We note that Hg pollution in the Xishui River is not considerable, with no Hg accumulation observed upstream of site 24, suggesting that the Chishui River is severely impacted by the anthropogenic activities in Hejiang County during its flow into the Yangtze River. A comparison of the average metal concentrations in the collected samples with the BV reveals that the sediments are contaminated. According to previous studies (Cai et al., 2017; Xu et al., 2020), anthropogenic activities are responsible for the high metal concentrations in the sediments of the Chishui River.

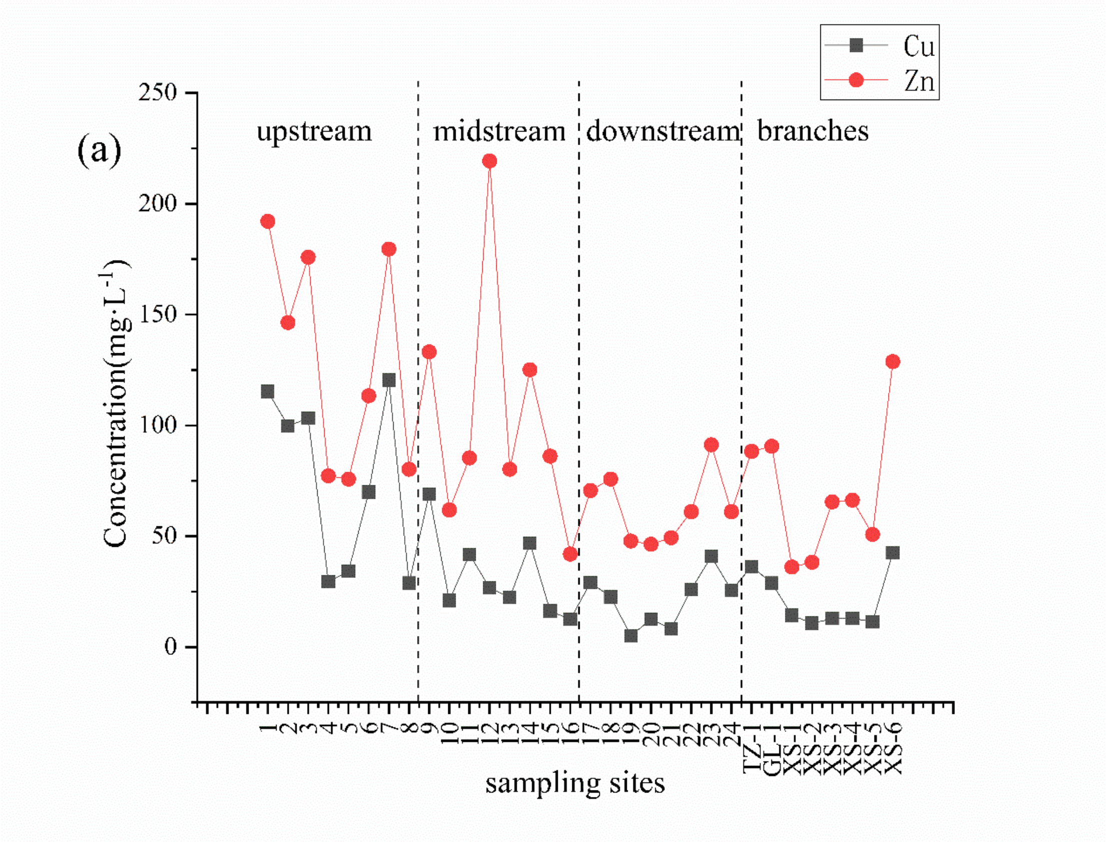
(a)
Cu
Zn
upstream
midstream
downstream
branches
Concentration(mg·L-1)
250
200
150
100
50
0
1 2 3 4 5 6 7 8 9 10 11 12 13 14 15 16 17 18 19 20 21 22 23 24 TZ-1 GL-1 XS-1 XS-2 XS-3 XS-4 XS-5 XS-6
sampling sites

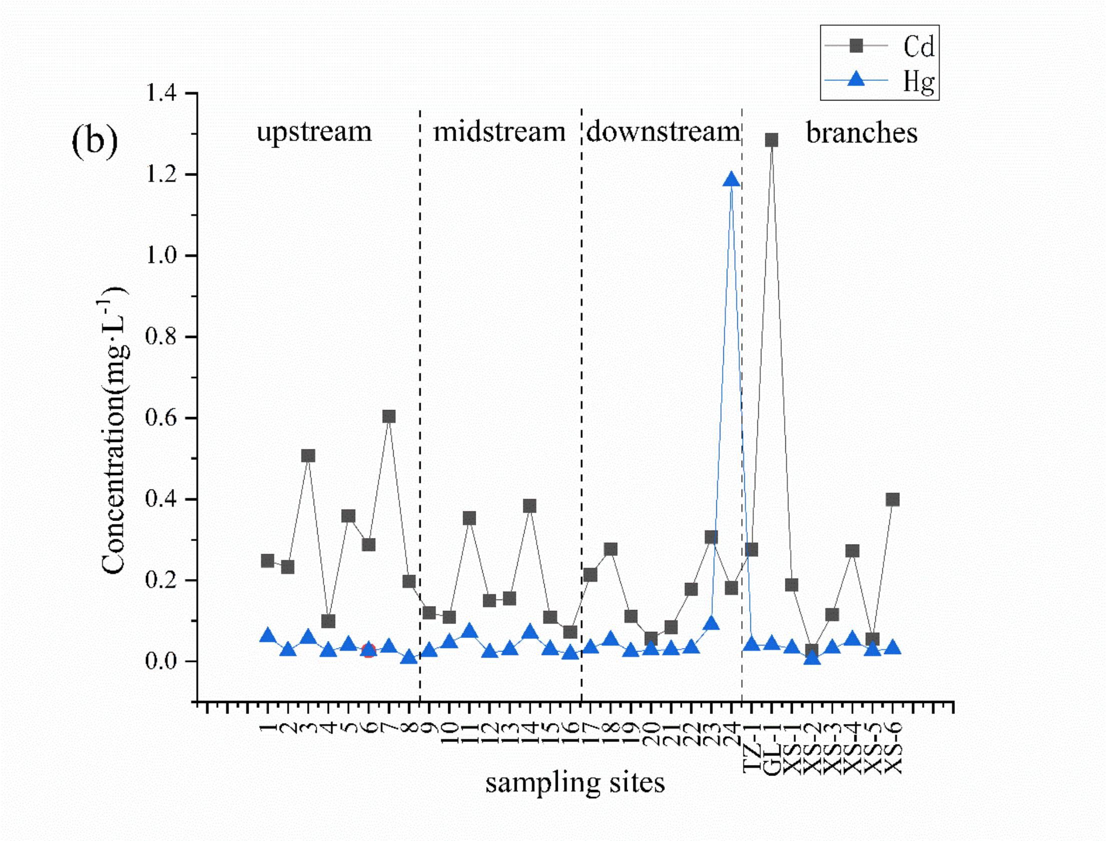
(b)
Cd
Hg
upstream
midstream
downstream
branches
Concentration(mg·L-1)
1.4
1.2
1.0
0.8
0.6
0.4
0.2
0.0
1 2 3 4 5 6 7 8 9 10 11 12 13 14 15 16 17 18 19 20 21 22 23 24 TZ-1 GL-1 XS-1 XS-2 XS-3 XS-4 XS-5 XS-6
sampling sites

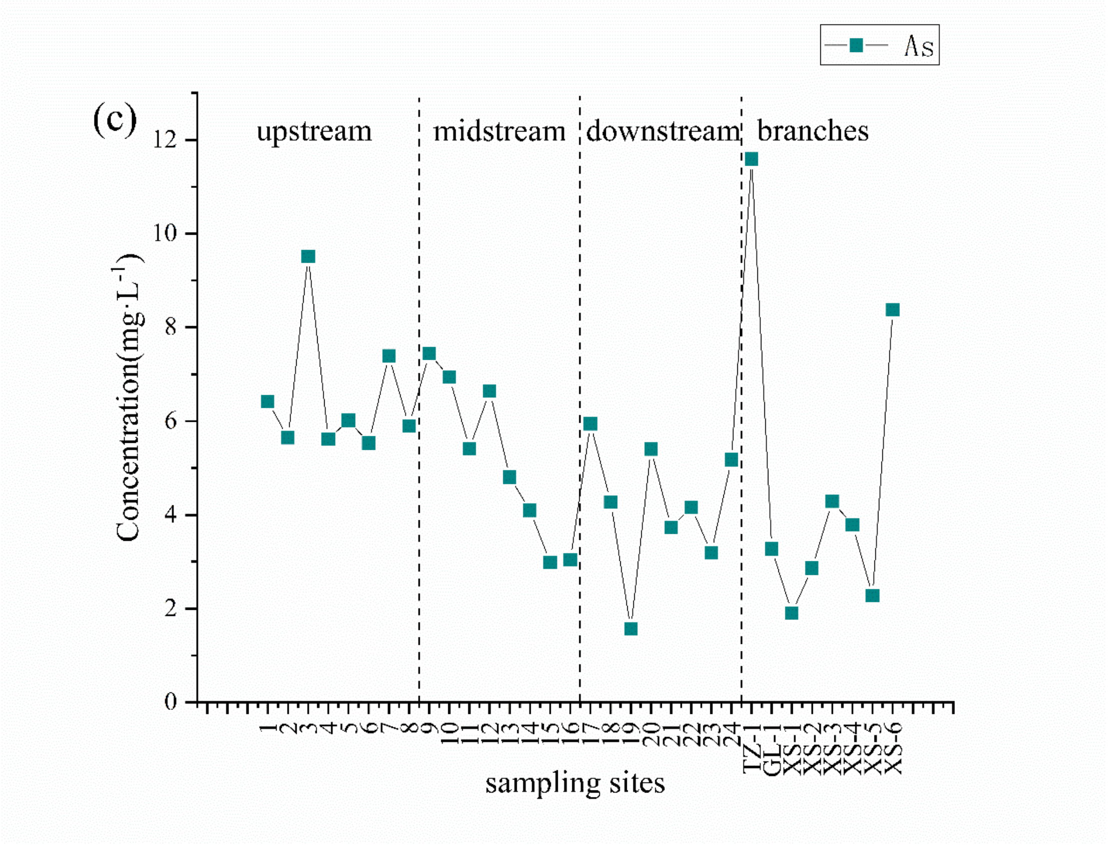

Fig. 2. Variation of measured heavy metals in the surface sediments of Chishui River Basin based on the direction of flow.

Table 4 Summary of Cu, Zn, Cd, Hg, and As concentrations in sediments collected from the Chishui River (mg kg^{-1}).

Sample no.	Cu	[illegible]	Cd	Hg	As
1	115.28	192.10	0.25	0.06	6.41
2	99.85	146.46	0.23	0.03	5.65
3	103.20	175.90	0.51	0.06	9.52
4	29.37	77.28	0.10	0.03	5.62
5	34.19	75.81	0.36	0.04	6.02
6	69.96	113.34	0.29	0.03	5.53
7	120.40	179.58	0.60	0.03	7.39
8	28.85	80.22	0.20	0.01	5.90
9	68.97	133.22	0.12	0.02	7.44
10	21.03	61.82	0.11	0.05	6.95
11	41.75	85.38	0.35	0.07	5.41
12	26.70	219.33	0.15	0.02	6.64
13	22.40	80.22	0.16	0.03	4.80
14	46.78	125.12	0.38	0.07	4.10

15	16.25	86.11	0.11	0.03	2.99
16	12.51	41.95	0.07	0.02	3.04
17	29.07	70.66	0.21	0.03	5.94
18	22.62	75.81	0.28	0.05	4.28
19	5.12	47.84	0.11	0.02	1.57
20	12.47	46.37	0.06	0.03	5.40
21	8.34	49.31	0.08	0.03	3.74
22	26.06	61.09	0.18	0.03	4.16
23	40.85	91.26	0.31	0.09	3.19
24	25.54	61.09	0.18	1.18	5.18
TZ-1	36.34	88.32	0.28	0.04	11.59
GL-1	28.85	90.53	1.28	0.04	3.28
XS-1	14.28	36.06	0.19	0.03	1.91
XS-2	10.75	38.27	0.03	0.01	2.87
XS-3	12.86	65.50	0.12	0.03	4.29
XS-4	12.94	66.24	0.27	0.05	3.79
XS-5	11.40	50.78	0.05	0.03	2.27
XS-6	42.66	128.80	0.40	0.03	8.38
Mean	37.43	91.93	0.25	0.07	5.16
Background value	21.5	73.6	0.15	0.034	7.6

A comparison of the data from this study with monitoring data from other areas in China (Li et al., 2010; Wang et al., 2012; Yan et al., 1995) reveals relatively lower heavy metal concentrations in the study area (Table 4). The concentrations of metals were lower than those of samples collected from the Tuojiang and East rivers, indicating that, owing to a reduced influence from industrialization in Guizhou Province relative to other parts of China, there is less riverine pollution.

Relative to the concentrations of heavy metals in rivers worldwide, Cu and Zn concentrations in the sediments from the Chishui River were correspondingly lower and higher than those of samples from the Swarnamukhi River Basin (India), whereas the Cd concentrations were comparable (Patel et al., 2018). Conversely, the Cu, Zn, Cd, and Hg concentrations for samples collected from the Chishui River were higher than

those of samples collected from the Halda River (Bangladesh) (Simul and Abu, 2017), with Cu and Cd concentrations approximately 6.3- and 6.2-fold higher, respectively. Additionally, sediments from the Chishui River exhibited lower Cd and higher As concentrations compared to those from the Thamirabarani River (India) (Ulaganathan et al., 2020). Furthermore, the Cu and Zn concentrations of the sediments from the Chishui River were 4.6- and 3.4-fold higher than those of sediments from the Hunza River, respectively (Pakistan) (Kashif et al., 2020). These differences in the concentrations of heavy metals between the sediments in this study and those from global rivers can be attributed to the sampling sites, levels of contamination, regional characteristics, and anthropogenic activities (Fabio et al., 2021).

Table 5 Concentrations (mg/kg) of the five heavy metals in the sediments collected from the Chishui River compared to other rivers in China and the world.

	Cu	Zn	Cd	Hg	As	Reference
Concentration range	5.12–120.40	36.01–219.31	0.03–1.28	0.01–1.18	1.56–11.59	This study
Mean	37.43	91.92	0.25	0.07	5.16	--
Median	27.77	78.80	0.19	0.03	5.29	--
Coefficient of variation	85.13 %	51.86 %	92.78 %	280.37 %	43 %	--
Background Value	21.5	73.6	0.15	0.034	7.6	(Alan, 1993)
Tuojiang	48.95	261	1.48	0.19	11.84	(Li et al., 2010)
East River	157.29	213.21	0.98	0.42	——	(Wang et al., 2012)
Mean concentration from rivers in China	21	68	0.14	0.042	9.1	(Yan et al., 1995)
Preindustrial reference value for lake sediments	50	175	1.0	0.25	15	(Hakanson, 1980)
Swarnamukhi River Basin	100.9	63.4	0.2	——	——	(Patel et al., 2018)
Halda River	5.9	79.58	0.04	0.001	——	(Simul and Abu, 2017)
Thamirabarani River	35.236	93.278	3.123	——	2.061	(Ulaganathan et al., 2020)
Hunza River	8	27	0.4	——	——	(Kashif et al., 2020)

3.2. Sediment contamination assessment

3.2.1. Geo-accumulation index assessment

Table 6 presents the results of the I_{geo} assessment, which highlights the extent of pollution associated with various metals. The I_{geo} values for the elements ranged from –2.66 to 1.90 for Cu (mean = –0.23), –1.61 to 0.99 for Zn (mean = –0.43), –3.11 to 2.51 for Cd (mean = –0.28), –3.24 to 4.54 for Hg (mean = –0.5), and –2.86 to 0.02 for As (mean = –1.28). The negative mean I_{geo} values for all elements indicate unpolluted areas. The I_{geo} value for Hg from sampling site 24 is an outlier, whereas 18.75 and 9 % of the sampling sites showed Cu and Cd accumulation, respectively. Moreover, upstream of the Chishui River, all heavy metals, except for As, were characterized by accumulation. The I_{geo} values reported in the present study for Cu, Zn, and Cd were higher than those reported by recent studies on similar sediments in other areas of China (e.g., Ren et al., 2021). The accumulation of the examined elements had the following order: Cu > Cd > Zn > Hg > As.

Table 6 I_{geo} values for five heavy metals in Chishui River sediments.

Sample no.	I_{geo}				
	Cu	Zn	Cd	Hg	As
1	1.84	0.80	0.14	0.25	–0.83
2	1.63	0.41	0.05	–0.96	–1.01
3	1.68	0.67	1.17	0.17	–0.26
4	–0.14	–0.51	–1.18	–1.01	–1.02
5	0.08	–0.54	0.67	–0.34	–0.92
6	1.12	0.04	0.35	–0.94	–1.04
7	1.90	0.70	1.43	–0.57	–0.62
8	–0.16	–0.46	–0.20	–2.69	–0.95
9	1.10	0.27	–0.91	–1.05	–0.62
10	–0.62	–0.84	–1.04	–0.16	–0.71
11	0.37	–0.37	0.65	0.51	–1.08
12	–0.27	0.99	–0.59	–1.24	–0.78
13	–0.53	–0.46	–0.54	–0.84	–1.25
14	0.54	0.18	0.77	0.46	–1.48
15	–0.99	–0.36	–1.05	–0.79	–1.93
16	–1.37	–1.40	–1.65	–1.42	–1.91

17	–0.15	–0.64	–0.08	–0.66	–0.94
18	–0.51	–0.54	0.30	0.05	–1.41
19	–2.66	–1.21	–1.03	–1.09	–2.86
20	–1.37	–1.25	–2.01	–0.85	–1.08
21	–1.95	–1.16	–1.41	–0.82	–1.61
22	–0.31	–0.85	–0.34	–0.65	–1.45
23	0.34	–0.27	0.44	0.83	–1.84
24	–0.34	–0.85	–0.32	4.54	–1.14
TZ-1	0.17	–0.32	0.29	–0.36	0.02
GL-1	–0.16	–0.29	2.51	–0.31	–1.80
XS-1	–1.18	–1.61	–0.25	–0.68	–2.58
XS-2	–1.58	–1.53	–3.11	–3.24	–1.99
XS-3	–1.33	–0.75	–0.96	–0.66	–1.41
XS-4	–1.32	–0.74	0.28	0.06	–1.59
XS-5	–1.50	–1.12	–2.05	–0.96	–2.33
XS-6	0.40	0.22	0.83	–0.69	–0.44
mean	–0.23	–0.43	–0.28	–0.50	–1.28

3.2.2. Pollution load index assessment

The PLI values of the 32 samples from the Chishui River Basin are shown in Fig.3. The values ranged from 0.31–2.47, with a median value of 1.09. According to these values, 9.37 % of the samples were considerably contaminated, 43.75 % were moderately contaminated, and 46.88 % were uncontaminated. The considerably contaminated samples were concentrated upstream of the Chishui River (samples 1, 3, and 7); among these, sample 3 showed the maximum PLI (Fig. 3). Mean PLIs of 1.40, 0.96, and 0.85 corresponded to the upstream, midstream, and downstream portions of the Chishui River, respectively, demonstrating its efficient self-purifying potential. The PLI values for the three branches had the following order: GL-1 (1.53) > TZ-1 (1.50) > XS1–X6 (0.8). The average PLI value of 1.8 for the entire basin indicates moderate contamination (PLI > 1).

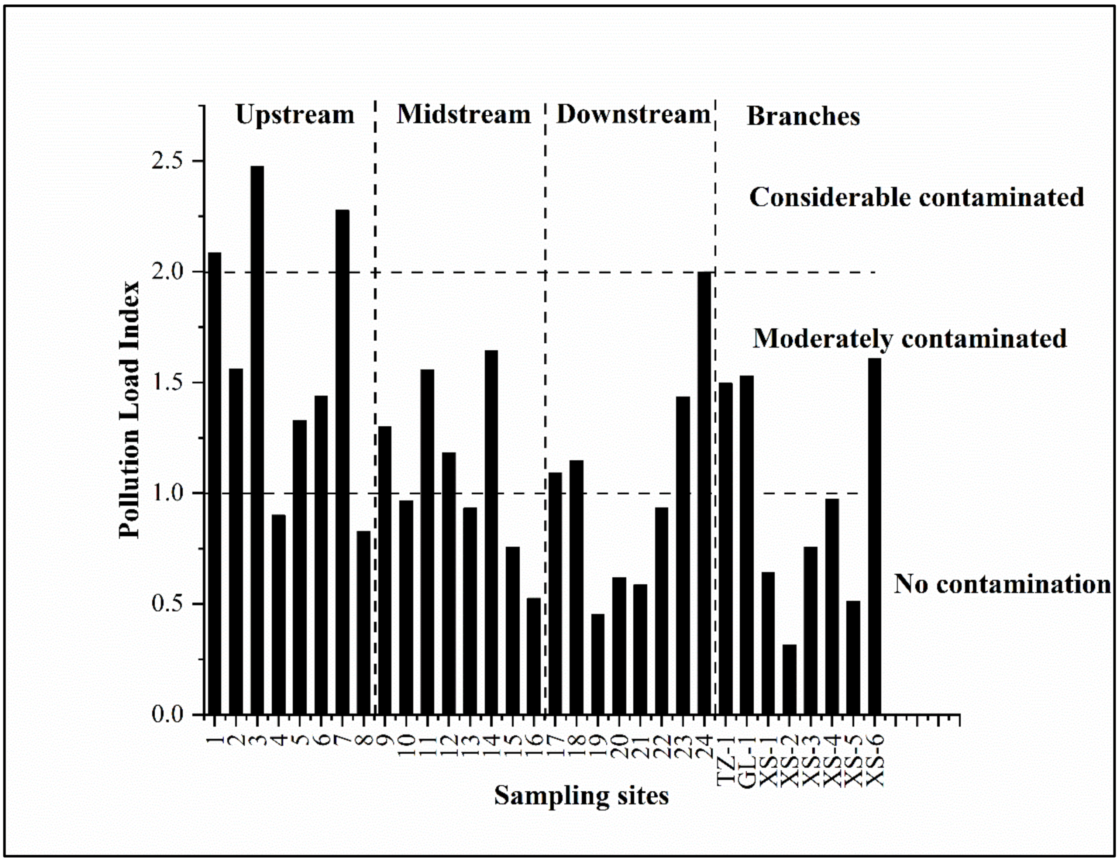

Fig. 3. Pollution load index for heavy metals in the sediments at different sites in the Chishui River Basin, China.

3.2.3. Potential ecological risk assessment

The Er(Ecological Risk) and RI values for Cu, Zn, Cd, Hg, and As in the sediments from the Chishui River Basin are presented in Table 7 and Fig. 4. The Er values ranged from 1.19–28 (mean = 8.79), 0.49–2.98 (mean = 1.25), 5.2–256.98 (mean = 50.04), 6.35–1393.29 (mean = 85.48), and 2.06–15.25 (mean = 6.79) for Cu, Zn, Cd, Hg, and As, respectively. The potential ecological risk associated with Hg was the highest among the heavy metals investigated in the surface sediments from the Chishui River Basin. The potential ecological risk index values ranged from 6.35 to 1,393.29, with a median value of 37.71, which suggests that most of the sampling sites have a low risk.

Approximately 36.88 % of the samples exhibited a moderate risk for Cd. However, the Er values of < 40 for Cu, Zn, and As for all 32 samples indicate a low ecological risk for these metals. Overall, the mean Er values for the five heavy metals in the sediments had the following order: Hg > Cd > Cu > As > Zn. The high ecological risk linked to sample 24 in Table 7 is mainly attributed to Hg. Although site 24 likely reflects point source pollution, data for 75 % of the investigated samples indicate a low ecological risk.

The five heavy metals produced RI values ranging from 18.34 to 1,443.02 (mean = 152.26), which represent low to very high ecological risks. The RI value of > 600 for sample 24 indicates a very high ecological risk site (the RI value of 1,443.02 for sample 24 showed in Fig. 4). The RI value for the 14 sites in the GL-1 section was > 300, which represents a considerable ecological risk; six of the 32 RI values were 150 ≤ RI ≤ 300, denoting moderate ecological risk; and 24 of the 32 RI values were < 150, implying a low ecological risk. Hg contributes significantly to the RI values because of its high toxicity and point source pollution, such as at site 24.

Table 7 Risk factors (E_r^i values) for heavy metals in sediments from the Chishui River Basin, China.

Site	E_r^i				
	Cu	Zn	Cd	Hg	As
1	26.81	2.61	49.58	71.53	8.44
2	23.22	1.99	46.54	30.82	7.43

3	24.00	2.39	101.36	67.53	12.52
4	6.83	1.05	19.82	29.88	7.39
5	7.95	1.03	71.58	47.29	7.92
6	16.27	1.54	57.40	31.29	7.28
7	28.00	2.44	120.84	40.35	9.73
8	6.71	1.09	39.30	9.29	7.76
9	16.04	1.81	24.02	28.94	9.79
10	4.89	0.84	21.86	53.76	9.14
11	9.71	1.16	70.62	85.29	7.12
12	6.21	2.98	29.96	25.41	8.74
13	5.21	1.09	31.00	33.53	6.32
14	10.88	1.70	76.68	82.71	5.39
15	3.78	1.17	21.74	34.59	3.93
16	2.91	0.57	14.36	22.35	4.00
17	6.76	0.96	42.64	38.00	7.82
18	5.26	1.03	55.36	62.00	5.63
19	1.19	0.65	22.10	28.24	2.06
20	2.90	0.63	11.20	33.29	7.11
21	1.94	0.67	16.88	33.88	4.92
22	6.06	0.83	35.52	38.24	5.48
23	9.50	1.24	61.24	106.35	4.20

24	5.94	0.83	36.16	1,393.29	6.81
TZ-1	8.45	1.20	55.16	46.59	15.25
GL-1	6.71	1.23	256.98	48.47	4.31
XS-1	3.32	0.49	37.74	37.53	2.51
XS-2	2.50	0.52	5.20	6.35	3.77
XS-3	2.99	0.89	23.14	37.88	5.64
XS-4	3.01	0.90	54.64	62.47	4.99
XS-5	2.65	0.69	10.86	30.94	2.99
XS-6	9.92	1.75	79.74	37.29	11.02

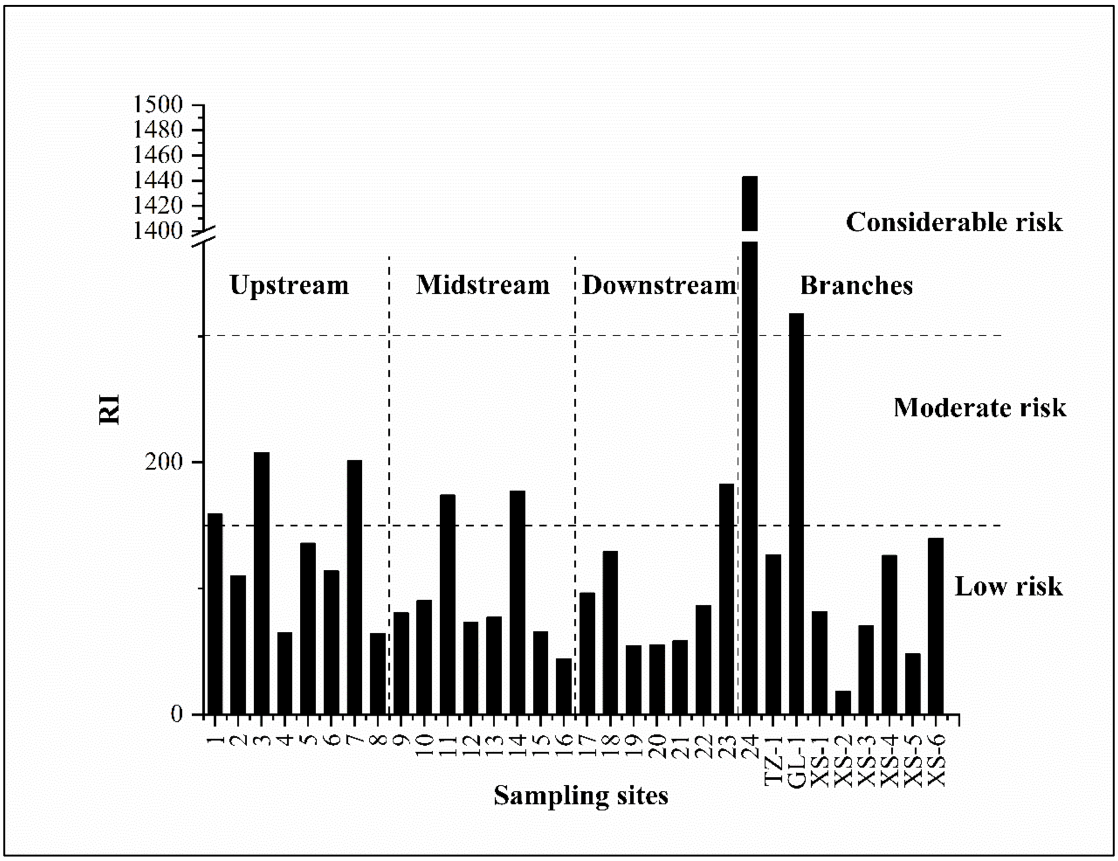

Fig. 4. Potential ecological risk indexes for heavy metals in sediments from the Chishui

River Basin, China.

3.3. Heavy metal source apportionment

The Pearson correlation matrix is useful for determining the sources and pathways of contaminants in river surface sediments (Bartoli et al., 2012). A correlation matrix for the elements studied is presented in Table 8. The confidence levels between Cu, Zn, and As were up to 99 %, which suggests similar pollution sources for these heavy metals. The weak positive correlations between Cd and the other heavy metals (except Cu) indicates that Cd is likely associated with contaminant sources different from those of Hg, Zn, and As. Moreover, the weak negative correlations between Hg and the other metals (excluding As) imply no relationships among these metals (Song et al., 2011).

Table 8 Pearson correlation coefficient matrix for the heavy metals in the Chishui River surface sediments.

	Cu	Zn	Cd	Hg	As
Cu	1				
Zn	0.796**	1			
Cd	0.351*	0.324	1		
Hg	–0.043	–0.098	–0.023	1	
As	0.538**	0.558**	0.154	0.009	1

Notes: ** Correlation is significant at the 0.01 level (2-tailed); * Correlation is significant at the 0.05 level (2-tailed).

4. Conclusions

The analysis of he[illegible]s (As, Cd, Cu, Hg, and Zn) in 32 sediment samples from the Chishui River Basin in the present study produced mean concentrations with the following order: Zn > Cu > As > Cd > Hg. The mean concentrations of Cu, Zn, Cd, and

Hg exceeded the corresponding BVs, whereas the concentration of As was lower than the BV. According to the I_{geo} values, 18.75 and 9 % of the sampled sites displayed Cu and Cd accumulation, respectively. The E_r^i values for the five heavy metals followed the order Hg > Cd > Cu > As > Zn, with 24 samples considered low Er, six being moderate Er, one being considerable Er, and one being very high Er. Hg contributed significantly to the RI values because of its higher toxicity. Overall, the Chishui River Basin is characterized by moderate contamination. According to a Pearson correlation matrix, sediment Cu, Zn, and As likely originate from similar contaminant sources. This study provides a reference for the formulation of policies in Guizhou. As the water source for Guizhou's wine industry, Chishui River is slightly polluted. In addition, Hg and Cd pollution in the Chishui River should be considered a serious problem.

Declaration of Competing Interest

The authors declare that they have no known competing financial interests or personal relationships that could have appeared to influence the work reported in this paper.

Acknowledgments

This study was funded by the Open Fund of the State Key Laboratory of Loess and Quaternary Geology [grant number SKLLQG2036], the Chinese Academy of Sciences Strategic Leading Science and Technology Project (Category B) [grant number XDB40000000], the Guizhou Institute of Technology High-level Talent Research Startup Funding Project [grant number 2019, 45], the Science and Technology Program

of Guizhou Province (Guizhou Science and Technology Cooperation Support [grant number 2018, 20101]), and the Science and Technology Foundation of Water Resources Department of Guizhou Province [grant number KT201508].

References

Alan, T., 1993. Metals and their compounds in the environment: occurrence, analysis and biological relevance. Elsev 271.

Awadh, A.K., Kong, Y.C., 2021. A review of heavy metals in coastal surface sediments from the red sea: health-ecological risk assessments. J. Int. J. Env. Res. Pub. He. 18.

Bartoli, G., Papa, S., Sagnella, E., Fioretto, A., 2012. Heavy metal content in sediments along the Calore river: relationships with physical–chemical characteristics. J. Environ. Manage. 95 Supplement, S9–14.

Cai, S., Ni, Z., Liu, B., Fan, L., 2017. Metal concentrations and health risk assessment in the muscle of ten commercial fish species from the Chishui River, China. Int J Environ Res 11, 125–132.

Cao, W., 2000. Thoughts on the construction of the endemic fish nature reserve in the upper reaches of the Yangtze River and related issues. Res Environ. Yangt Bas, 131–132.

Fabio, F.-G., José, P.-H., Edwin, G., José, M.-N., Sergi, D., 2021. Heavy metal pollution and toxicity assessment in Mallorquin swamp: a natural protected heritage in the Caribbean Sea, Colombia. Mar. Pollut. Bull. 167:112271.

Fang, M., Wu, Y., Liu, H., Jia, Y., Zhang, Y., Wang, X., Wu, M., Zhang, C., 2013. Distribution, source and potential ecological risk assessment of heavy metals in sediments of the Yangtze River Estuary. J. Environ. Sci. 33, 563–569.

Hakanson, L., 1980. An ecological risk index for aquatic pollution control. A sedimentological approach. Water Res. 14, 975–1001.

Herath, D., Pitawala, A., Gunatilake, J., Iqbal, M.C.M., 2018. Using multiple methods to assess heavy metal pollution in an urban city. Environ. Monit. Assess. 190, 657.

Huang, Z., 2003. On the development and conservation for the resources and ecology of Chishui River basin. Res Environ. Yangt Bas 12, 332–339.

Ji, Y., Fang, H., Shen, X., Sheng, H., Zhao, Q., 2012. Trend of water environment quality in Maotai section of the Chishui River. GZ Chem. Ind. 5.

Jiang, H., An, Y., Wu, Q., Luo, J., Peng, W., 2013. Study on spatial distribution of water quality in Chishui River basin in dry season, The 14th Annual Conference of Chinese Society of Mineralogy. Petrology and Geochemistry, Nanjing, Jiangsu, China, p. 1.

Kashif, A., Said, M., Wajid, A., Ishtiaq A.K, J., Atta, R., 2020. Occurrence, source identification and potential risk evaluation of heavy metals in sediments of the Hunza river and its tributaries, Gilgit-Baltistan. Environ. Technol. Innov.

Kükrer, S., Şeker, S., Abacı, Z.T., Kutlu, B., 2014. Ecological risk assessment of heavy metals in surface sediments of northern littoral zone of Lake Çıldır, Ardahan, Turkey. Environ. Monit. Assess. 186, 3847–3857.

Li, J., Shi, Z., Zheng, L., Ni, S., 2010. Potential ecological risk assessment of heavy metals in sediments of Tuojiang river basin. Earth Environ. 38, 481–487.

Luo, J., An, Y., Wu, Q., Yang, R., Jiang, H., Peng, W., Yu, X., Lv, J., 2014. Analysis of the spatial distribution characteristics of river water chemistry in the middle and lower reaches of Chishui River in winter. Earth Environ. 42, 297–305.

Lv, J., An, Y., Wu, Q., Luo, J., Jiang, H., Peng, W., 2013. Study on the hydrochemical characteristics of the upper Chishui River basin in the dry season, The 14th Annual Conference of Chinese Society of Mineralogy. Petrology and Geochemistry, Nanjing, Jiangsu, China, p. 1.

Malvandi, H., 2017. Preliminary evaluation of heavy metal contamination in the Zarrin-Gol river sediments, Iran. Mar. Pollut. Bull. 117, 547–553.

Muller, G., 1969. Index of geoaccumulation in sediments of the Rhine River. Geol. J. 2, 108–118.

Patel, P., Raju, N.J., Reddy, B.C.S.R., Suresh, U., Sankar, D.B., Reddy, T.V.K., 2018. Heavy metal contamination in river water and sediments of the Swarnamukhi river basin, India: risk assessment and environmental implications. Environ. Geochem. Health. 40, 609–623.

Qiu, L., Zheng, Y., Zhai, H., Fan, H., 2013. Research on ecological compensation mechanism of Chishui River basin. People's. Yangtze River 44, 94–96 + 108.

Ren, J., Bai, L., Li, J., Hu, J., Hu, X., 2021. Evaluation and source analysis of heavy metal pollution in the surface sediments of Taihu Lake. Earth Environ., 1–12.

Ren, X., Huang, M., 2009. Industry status and strategy of integrated river basin management in the Chishui River Basin. Res Environ. Yangt Bas 02.

Islam, M.S., Ahmed, M.K., Habibullah-Al-Mamun, M., Masunaga, S., 2015. Assessment of trace metals in fish species of urban rivers in Bangladesh and health implications. Environ. Toxicol. Pharmacol. 39, 347–357

Simul, B.M., Abu, B.M., 2017. Seasonal variation of heavy metals in water and sediments in the Halda river, Chittagong, Bangladesh. Environ. Sci. Pollut. Res. Int. 24.

Song, Y., Ji, J., Yang, Z., Yuan, X., Mao, C., Frost, R.L., Ayoko, G.A., 2011. Geochemical behavior assessment and apportionment of heavy metal contaminants in the bottom sediments of lower reach of Changjiang River. CATENA 85, 73–81.

Suresh, G., Sutharsan, P., Ramasamy, V., Venkatachalapathy, R., 2012. Assessment of spatial distribution and potential ecological risk of the heavy metals in relation to granulometric contents of Veeranam lake sediments, India. Ecotoxicol. Environ. Saf. 84, 117–124.

Ulaganathan, A., Jeya, S.R., Rajendran, S., Geevaretnam, J., 2020. Human health risk assessment of heavy metals in aquatic sediments and freshwater fish caught from Thamirabarani River, the Western Ghats of South Tamil Nadu. Mar. Pollut. Bull.

159.

Wang, A., 2011. Primary probe into geographical environment and culture symbiosis of Chishui River valley. J. Anh Agric. Sci. 31.

Wang, F., Hong, H., Cen, Y., Chen, Y., 2012. Analysis of heavy metals in sediment of Dongjiang river basin and potential environmental ecological risks. Environ. Chem. 31, 1328–1334.

Wedepohl, K., Merian, E., 1991. Metals and Their Compounds in the Environment-Occurrence Analysis and Biological Relevance, Weihein (Federal Republic of Germany).

Wu, J., Zhao, H., Miao, Z., Chen, Y., Zhang, F., Wang, J., 2010. Status and conservation of fish resources in the Chishui River. Biodivers. Sci. 18, 162.

Wu, Z., 2001. The Water Environment Background Value and Geochemical Characteristics of Chishui River System. Guiz Environ Prot Tech, pp. 25–30.

Xu, S., Lang, Y., Zhong, J., Xiao, M., Ding, H., 2020. Coupled controls of climate, lithology and land use on dissolved trace elements in a karst river system. J Hydrol 591, 125328.

Yan, M., Chi, Q., Gu, T., Wang, C., 1995. Average content of chemical elements in various sediments in China. Geophys. Geochem. Explor., 468–472.

Zhen-li, H., 2003. on the development and conservation for the resources and ecology of Chishui river basin. Resour. Environ. Yangtze Basin 12, 332–339.

Zou, F., 2010. Status of water environment quality in the middle part of the Chishui River, Chin Pop. Resour. Environ. S 1.

Zoumis, T., Schmidt, A., Grigorova, L., Calmano, W., 2001. Contaminants in sediments: remobilisation and demobilisation. Sci. Total Environ. 266, 195–202.

Highlights

Click here to access/download
Supporting Information
Highlights.docx
