# Supplementary material for: Assessment of heavy metal pollution in surface sediments of the Chishui River Basin, China
Source: PLoS One. 2022 Feb 9;17(2):e0260901. doi: 10.1371/journal.pone.0260901 (PMC8827479; doi:10.1371/journal.pone.0260901)
Supplement: Attachment — Submitted filename: Response to Reviewers.docx [file pone.0260901.s003.docx]

**Authors response**

Reviewer #1:

**1.** In page #6, the authors should verify the equation: = log2[ /(1.5 × )]. If necessary,“i” may be replaced with “n”.

Response：We are grateful to Reviewer 1 for the positive comments on our manuscript. According to your advice, we have replaced “i” with “n”.

**2.** It appears that the authors wanted to cite specific references for each equation but it is not in proper format. Please see below for an example and edit as needed. Page#6: = log2[ /(1.5 × )], (1)

Response: We have edited the format of all formula references

3. Figure 2 (a,b,c) should be compiled for one figure (Page 12 and 13)

Response: we have compiled Figure 2 (a,b,c) for one figure.

4. The authors missed inserting line numbers in the whole manuscript.

Response: now we have inserted line numbers in the whole manuscript.

5. The authors should write completely for different abbreviations. For example, in line # 3 of page 3 pollution load should be changed to “pollution load index. Similarly, RI should be replaced with ERI (line # 3 of Page #3).

Response: we carefully correct all the mistakes you noted in the manuscript.

Reviewer #2:

1. This paragraph is suggested to move to the end of the Introduction part

Response: We are grateful to Reviewer 2 for the professional comments on our manuscript. According to your advice, we have moved this paragraph to the end of the Introduction part.

2. These detailed information on the study site can be moved to the method and materials, while only keep simple description on Chishui here to raise the scientific questions

Response: we have moved the detailed information to the method and materials and keep simple description on Chishui to raise the scientific questions.

3. Are there any studies to prove that the species are threatened by the contaminants.

Response: We have added corresponding research on the impact of species of Chishui River. Human activities cause the decline of fish resources in Chishui River (Wu, 2010)

4. This paragraph described several related studies, however, only the content or objective of the studies were described, and no results or conclusion were mentioned. The results of previous studies should be well summarized to show how the contamination condition are for the Chishui River and why it is necessary to investigate the heavy metals. The lack or limitation of relative studies were not the proofs for the necesity to conduct the heavy metals study.

Response: We have summarized the previous research results to explain the pollution status of Chishui River and prove the necessity of heavy metal research. Previous studies have focused on the development and protection of cultural, touristic, and natural resources along the Chishui River, limited studies have examined heavy metal pollution and their spatial distributions in the sediments along the entirety of the Chishui River Basin, so this study may be useful for future investigations on Chishui River.

5. How many samples were collected from each site?

Response: we collected 1 sample from each site in the 0-10 cm layer, and every site was mixed by several sediments near the sampling location.

6. As mentioned above, the detailed information of study sites should be described here and the number and distribution of the stations also need to be explained, especially the classification of upstream, midstream and downstream. Please reorganize this paragraph.

Response: We have added more details about the classification of upstream, midstream and downstream. 19 sites were on the main stream and other 13 sites were on the tributaries, which basically covered the entire basin of the Chishui River. 1-8 sampling sites were located at upstream, sites 9-16 were distributed in the middle stream and sites 17-24 were at downstream.

7. The specific statistical method should be listed here.

Response: we have listed the specific statistical method we used in this research at line 115, Pearson correlation matrix were calculated for measured elements separately to identify the similaritie**s**.

8. Table 4 showed the same data as Figure 2, it can be deleted.

Response: We have removed Table 4 from the manuscript.

9. All the results only contain 1 value and it can not be compared using statistical method, thus, the results might not trustful. The results and discussion part was moslty the description of current study and lack of the comparison among different studies, especially the comparison with other areas in China or similar river and also lack of explanation on the origin of heavy metals and potential risk on animals or human being.

Response: According to your advices, we have added comparisons with other rivers in China and analyzed the sources of heavy metals.

10. The conclusion part was a repetition of the results part, it should be reorganized to summarize the important finding and the implication

Response: we have reorganized to summarize the important finding and the implication.
