# Supplementary material for: Assessment of heavy metal pollution in surface sediments of the Chishui River Basin, China
Source: PLoS One. 2022 Feb 9;17(2):e0260901. doi: 10.1371/journal.pone.0260901 (PMC8827479; doi:10.1371/journal.pone.0260901)
Supplement: Attachment — Submitted filename: Response to Reviewers.docx [file pone.0260901.s004.docx]

**Authors response**

Reviewer #1:

**1.**It appears that the authors have not edited the reference format for the equations (Line # 155, 168, 169, 170, 184, 190,

Response：We are grateful to Reviewer 1 for the positive comments on our manuscript. According to your advice, we reedited the equations to make sure it fits the reference format.

**2.** Many references are missing. For example, Wu (2001) (Line #71) and Zou et al (2010) (# 76). The authors should check carefully throughout the manuscript.

Response：We have added the missing references.

**3.** The authors should check carefully grammatical errors throughout the manuscript (e.g., line #108 – 112)

Response：We have corrected the grammatical errors throughout the manuscript.

Reviewer #2:

Response：Thank you very much for your careful review to our manuscript.
